# pH-Dependent Adsorption of Peptides on Montmorillonite for Resisting UV Irradiation

**DOI:** 10.3390/life10040045

**Published:** 2020-04-20

**Authors:** Rongcan Lin, Yueqiao Wang, Xin Li, Yan Liu, Yufen Zhao

**Affiliations:** 1 Key Laboratory for Chemical Biology of Fujian Province, Department of Chemical Biology, College of Chemistry and Chemical Engineering, Xiamen University, Xiamen 361005, China; lyric@stu.xmu.edu.cn (R.L.); qiao510614851@163.com (Y.W.); 13606093461@163.com (X.L.); yfzhao@xmu.edu.cn (Y.Z.); 2 Institute of Drug Discovery Technology, Ningbo University, Ningbo 315211, China; 3 Key Laboratory of Bioorganic Phosphorus Chemistry and Chemical Biology (Ministry of Education), Department of Chemistry, Tsinghua University, Beijing 100084, China

**Keywords:** pH-dependent, adsorption, peptides, UV radiation protection, montmorillonite

## Abstract

Ultraviolet (UV) irradiation is considered an energy source for the prebiotic chemical synthesis of life’s building blocks. However, it also results in photodegradation of biology-related organic compounds on early Earth. Thus, it is important to find a process to protect these compounds from decomposition by UV irradiation. Herein, pH effects on both the adsorption of peptides on montmorillonite (MMT) and the abilities of peptides to resist UV irradiation due to this adsorption were systematically studied. We found that montmorillonite (MMT) can adsorb peptides effectively under acidic conditions, while MMT-adsorbed peptides can be released under basic conditions. Peptide adsorption is positively correlated with the length of the peptide chains. MMT’s adsorption of peptides and MMT-adsorbed peptide desorption are both rapid-equilibrium, and it takes less than 30 min to reach the equilibrium in both cases. Furthermore, compared to free peptides, MMT-adsorbed peptides under acidic conditions are well protected from UV degradation even after prolonged irradiation. These results indicate amino acid/peptides are able to concentrate from aqueous solution by MMT adsorption under low-pH conditions (concentration step). The MMT-adsorbed peptides survive under UV irradiation among other unprotected species (storage step). Then, the MMT-adsorbed peptides can be released to the aqueous solution if the environment becomes more basic (releasing step), and these free peptides are ready for polymerization to polypeptides. Hence, a plausible prebiotic concentration–storage–release cycle of amino acids/peptides for further polypeptide synthesis is established.

## 1. Introduction

The origin of life remains an unsolved mystery, because none of us can go back to the time when life first emerged [1]. In order to provide a reasonable explanation of the beginning of life, many scientists have made tremendous efforts to mimic prebiotic conditions (spark discharge, UV irradiation, shock waves, etc.) to investigate how the key components (amino acids, peptides, and nucleic acids) of living cells could have been produced [2]. In 1953, the Miller–Urey experiment confirmed that amino acids were formed by discharging a reducing atmosphere [3]. Amino acids have also been found in meteorites, among other organic compounds [4]. Combining all these results, amino acids have been considered relatively abundant substrates on early Earth. Later, the formation of oligopeptides by amino acid polymerization is suggested as a crucial step in the chemical evolution of life [4]. This process in aqueous solution is thermodynamically unfavored, because their standard free energy formation value is around 2.5~3.6 kcal/mol [5]. However, starting from amino acids and/or their derivatives, oligopeptide formation has been successfully achieved under plausible prebiotic conditions [6,7,8,9,10,11]. Recently, Powner and his co-workers have reported that peptides can form without amino acids. They found that aminonitriles, precursors of amino acid, can be turned into peptides in water with more ease and chemoselectivity than amino acids [12]. 

It is suggested that the UV flux on the surface of early Earth (four billion years ago) was approximately one order of magnitude higher than today due to the absence of an ozone layer [13,14]. Prebiotic building blocks, such as amino acids [15,16,17], nucleotides [17,18,19] and lipids [17] are synthesized under UV irradiation. Furthermore, iron–sulfur clusters, which are ancient cofactors playing a fundamental role in metabolism, are generated by UV irradiation as well [20]. It is worth mentioning that irradiation time is a key parameter for all experiments [17]. Prolonged UV irradiation can destroy prebiotic organic compounds [21,22]. Scappini and his co-workers simulated the photodegradation of phenylalanine (Phe), tryptophan (Try) and tyrosine (Tyr) in aqueous solution. They found that the concentration of these amino acids decreased to very low levels after a long irradiation [23,24]. To avoid prebiotic building block photodegradation, they have proved that the adsorption of nucleic acid and RNA-type molecules on montmorillonite (MMT) helps them survive UV irradiation [25]. MMT, which is common clay on early Earth, is usually formed by the reaction of volcanic ash with water [26]. MMT has a layered structure, which includes high valence cations (Si^4+^, Al^3+^, Fe^3+^) in the octahedral sheets, which can be replaced by lower valence cations (such as Mg^2+^ and Na^+^). Due to this ion exchange property, it is able to adsorb amino acids [27], peptides [28], nucleic acid bases [29] and ribose [29]. Meanwhile, some important monomers are selected and concentrated from dilute solution by MMT, and they are then prompted to form biopolymers [30]. MMT can also catalyze a variety of organic reactions that are critical to the origin of life [28]. Since MMT could potentially play some important roles in the chemical evolution of life, it is of great interest and significance to further investigate whether MMT can protect peptides under UV irradiation and what the related protective mechanism is.

According to Bernal’s suggestion in 1951, the formation of complex molecules must have required the presence of a protected, confined environment, namely a clay-rich setting. In these environments, biomolecules could originate, accumulate, and evolve while being protected from UV irradiation [31]. Under UV irradiation, peptides can be broken up easily, especially under prolonged irradiation [32]. The effects of UV irradiation on Gly-Tyr aqueous solutions were studied by Scappini et al. They found that Gly-Tyr decomposed rapidly under UV irradiation [33]. Hence, it is important to understand how peptides can survive under UV irradiation in the prebiotic environment. Although the adsorption of some peptides (Gly_2_, Gly_3_, Gly_4_, Gly-Ala) on MMT has been investigated [27,28], important questions remain unclear. Does MMT adsorb other peptides? Does the adsorption efficiency change at different pH values? Can MMT protect peptides from photodegradation under UV irradiation? Therefore, the present study aimed to systematically explore the effects of UV irradiation on free amino acid/peptides and MMT-adsorbed amino acid/peptides under different pH values. Phenylalanine (Phe) and six dipeptides (Phe_2_, Tyr_2_, Val_2_, Leu_2_, Pro_2_ and Ala_2_) were selected for our studies. These samples contain dipeptides with aromatic rings (Phe_2_, Tyr_2_) as well as dipeptides without aromatic rings (Val_2_, Leu_2_, Pro_2_ and Ala_2_). In order to further explore how adsorption rate and protection efficiency are influenced by the length of the peptide, a pentapeptide FFFFD, which is a fragment coming from one of the ancient proteins, namely ATP binding protein [34], was also used in this study.

## 2. Materials and Methods

### 2.1. Materials

**Figure 1:**
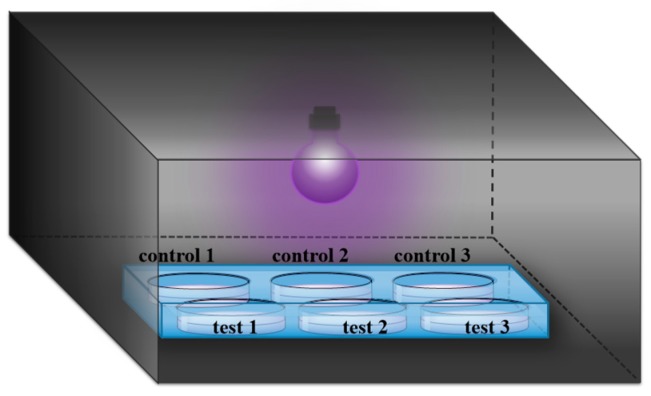
UV irradiation reaction device. Controls 1, 2 and 3 are three parallel control groups of the tested sample without MMT. Tests 1, 2 and 3 are three parallel experimental groups of the tested sample with MMT.

Phe was purchased from GL Biochem Ltd. (Shanghai, China). Phe_2_, Tyr_2_, Val_2_, Pro_2_, Leu_2_ and Ala_2_ were obtained from Synpeptide (Nanjing, China). The peptide FFFFD (purity: 95%) was purchased from Sangon Biotech (Shanghai, China). Unless otherwise noted, these amino acid residues are L-configuration. HPLC grade acetonitrile (ACN) was purchased from Fisher Scientific (Loughborough, UK). Formic acid (FA, as eluent additive for HPLC-MS) was purchased from Sigma-Aldrich Chemical Co. (Shanghai, China). Montmorillonite (MMT) with a 240-m^2^/g specific surface area was obtained from Aladdin Co. Ltd. (Shanghai, China). All chemicals were used as received without further purification. All solutions were prepared using ultrapure water obtained from a Millipore Milli-Q water purification system (Billerica, MA, USA) with an electric resistance > 18.2 MΩ. UV light was produced from a mercury lamp (Cnlight, Foshan, China), with a primary energy at 254 nm (28 μW/cm^2^). The distance between the sample and the UV lamp was about 18 cm (Figure 1).

### 2.2. Sample Preparation

Phe_2_ (500 μM), Tyr_2_ (500 μM), Leu_2_ (500 μM), Pro_2_ (500 μM), FFFFD (500 μM), Phe (500 μM), Ala_2_ (1 mM) and Val_2_ (2 mM) were prepared as stock aqueous solutions. For the adsorption experiments, MMT (0.2 g) was added to 2 mL peptide solution in a 2 mL centrifuge tube and then sodium hydroxide (6 M) or hydrochloric acid (6 M) was used to adjust the pH to the reported value. The suspensions were then agitated with a rotating mixer at room temperature for a designed time to achieve the adsorption equilibrium. After that, the samples were centrifuged for 2 min at 10,000 rpm to separate MMT residue and supernatant. The adsorption equilibrium was estimated by LC-MS analysis when the concentration of peptide in the supernatant no longer changed.

For adsorption efficiency analysis at pH 3, after removing the supernatant of the centrifuged MMT samples, the resulting samples were resuspended with 1 mL deionized water, and then the pH was adjusted from 3 to 10 with 6 M sodium hydroxide to desorb peptides from MMT. The resulting supernatants were centrifuged for 2 min at 10,000 rpm and filtrated through a 0.22 μm pore size filter for the analysis by LC-MS.

On the basis of Sasselov and coworker’s report [35] that 254 nm UV light is unshielded by H_2_O and available across a broad range of CO_2_ concentrations in the atmosphere, 254 nm UV light is a suitable light source for initial studies on prebiotic chemistry. Therefore, a mercury lamp which is able to generate 254 nm UV light was selected as the UV light source. For UV irradiation experiments, 2 mL of the solutions (pH = 3 or 10) with and without MMT were placed in a 6-well plate and dried at 55 °C. Then, the dried samples were placed in a non-airtight cassette with UV light as shown in Figure 1. After 1 day or 5 days of irradiation, the resulting samples were resuspended with water (2 mL) for the subsequent desorption and quantitative analysis.

Based on the previous report method [25,30], the above UV irradiation experiments were carried out under the natural aerobic environment. UV surface flux is insensitive to plausible levels of O_2_ [35], which means the presence of O_2_ cannot affect the intensity of UV radiation. However, O_2_ can produce some radicals under UV irradiation. Our main aim here is to reveal the adsorption and protection capacity differences of MMT for amino acid or dipeptides under varying pH conditions. Therefore, we set up a parallel control group without the addition of MMT, then oxygen interference could be offset as the background. The estimated total irradiances for 1 day and 5 days were 24.192 kJ/m^2^ (28 × 10^−2^ W/m^2^ × 1 × 24 × 3600s = 24.192 kJ/m^2^) and 120.96 kJ/m^2^ (28 × 10^−2^ W/m^2^ × 5 × 24 × 3600s = 120.96 kJ/m^2^), respectively. The UV lamp delivers about 2.25-fold higher flux to the sample than the natural environment [36].

For the samples under acidic conditions, sodium hydroxide (6 M) was used to adjust the pH from 3 to 10 to desorb peptides from MMT. The desorbed peptides were quantified using high-performance liquid chromatography (HPLC) as described below. The survival rate was determined through quantitative analysis of residual tested peptides.

All experiments were carried out three times in parallel. All quantitative analyses were performed on the basis of the linearity curve of standard peptides by HPLC (Figures S1–S8 in Supporting Information).

### 2.3. HPLC-MS Methods

Based on the different properties of the samples, two elution conditions were used with the same flow rate of 0.43 mL·min^−1^. Method 1 (suitable for Phe, Phe_2_, Val_2_, Tyr_2_, Pro_2_, Leu_2_ and FFFFD): a binary mobile phase (A: 0.1% formic acid in water; B: acetonitrile) was used. The gradient elution program was as follows: 0 min, 5% of B; 22 min, 50% of B; 24 min, 50% of B; 28 min, 5% of B; 33 min, 5% of B. Method 2 (suitable for Ala_2_: a binary mobile phase (A: 2 mM nonafluoropentanoic acid (NFPA) in water; B: acetonitrile) was used. The isocratic elution program was as follows: 96% of A in 20 min. MS instrument parameters were as follows: capillary voltage, 4500 V; nebulizer pressure, 2 bar; dry gas, 8 L·min^−1^; dry temperature, 200 °C. Mass spectra were recorded in positive mode with a scan range from *m/z* 50 to 1000. For ESI-MS analysis, about 1/2 of the eluate from LC was introduced to an ESI source through a splitting T valve.

### 2.4. X-Ray Diffraction (XRD) Analysis

1 mL Pro_2_ (10 mM) solution was mixed with MMT (0.1g) at room temperature at pH 3 for 0.5 h. The mixture was then dried at 55 °C. In order to avoid the influence of water molecules between layers of MMT on XRD measurement, the samples were solvated with ethylene glycol (EG) under vapor pressure at 60 °C for 24 h and after that immediately analyzed by XRD. Subsequently, MMT and MMT-adsorbed Pro_2_ were placed on a glass slide; XRD patterns were recorded on a Rigaku-Ultima IV XRD (Japan) equipped with Cu Kα working at 40 kV and 30 mA. The diffraction peaks were scanned between 4° and 20° with a scan speed of 5°/min. The raw data were smoothed in Origin 8.5 with the Savitzky–Golay method.

## 3. Results

### 3.1. Adsorption of Peptides on MMT under Different pH Conditions

**Figure 2:**
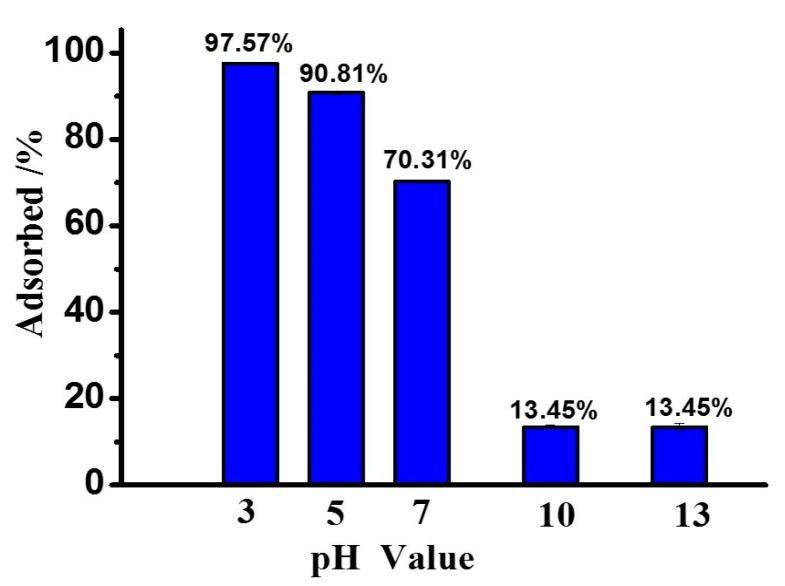
pH-dependent adsorption profile of Phe_2_ on MMT.

The adsorption rates of peptides on MMT at different pH values (3, 5, 7, 10 and 13) were studied using Phe_2_ as a model peptide. As shown in Figure 2, the absorption rate of Phe_2_ on MMT in an aqueous phase system was based on the pH value. Under acidic conditions (pH 3), 97.8% of Phe_2_ was adsorbed on MMT. Under basic conditions (pH 10 or 13), the adsorption rate dropped to 13.5% in both cases. These results indicated adsorption of Phe_2_ on MMT was preferred under acidic conditions rather than basic conditions. To monitor the absorption–desorption equilibrium of Phe_2_ on MMT by changing pH value, a sample was prepared at pH 3. After the equilibrium point was reached, the pH value of this sample was increased to 10 by adding sodium hydroxide (6 M), and then the supernatant was analyzed by HPLC-MS again. It was observed that 88.4% of Phe_2_ was desorbed from MMT. Thus, the adsorption/desorption of peptides on MMT is a dynamic process related to pH value.

In order to evaluate the adsorption efficiency of peptides with different amino acid residues and different lengths of peptides, adsorption and desorption experiments for the other peptides/amino acids (Tyr_2_, Val_2_, Ala_2_, Leu_2_, Pro_2_, FFFFD and Phe) on MMT were conducted at pHs 3 and 10. Adsorption and desorption were allowed to proceed for 30 min. All the results are summarized in Table 1. At pH 3, the adsorption rates of peptides and amino acids on MMT were much greater than that at pH 10. All the tested samples were adsorbed well by MMT at pH 3, with varying adsorption efficiency. The adsorption rates of Phe_2_, Pro_2_ and FFFFD on MMT were more than 90%. FFFFD had the highest adsorption rate (99.4%). The adsorption rate of Phe by MMT was the lowest, at only 47.5%. Comparing the adsorption rates of Phe, Phe_2_, and FFFFD, all of them contained Phe, and the adsorption rates of these substances on MMT gradually increased as the peptide chain extended. Additionally, all the peptides/amino acids tested here were more effectively adsorbed on MMT at pH 3 than at pH 10. All tested peptides/amino acids were desorbed from MMT by increasing the pH value to 10, with a desorption rate of more than 63%. These results indicate the adsorption/desorption process was a pH-dependent process. Furthermore, the adsorption of peptides/amino acids on MMT was universal and was positively correlated with the peptide chain length.

**Figure 3:**
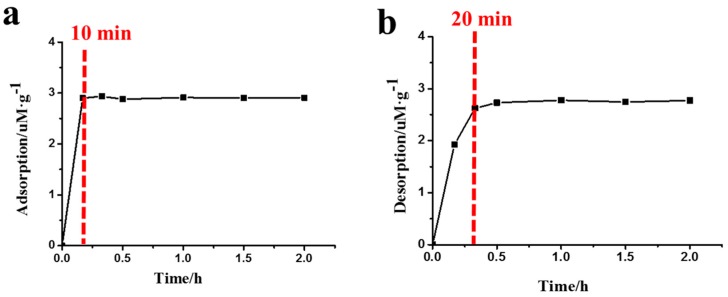
The time curves of adsorption at pH 3 (**a**) and desorption at pH 10 (**b**) of Phe_2_ on MMT.

Figure 3 illustrates the amount of Phe_2_ adsorption/desorption on MMT at different times. At pH 3, it was found that the adsorption of Phe_2_ on MMT reached an equilibrium state after 10 min (Figure 3a), suggesting the adsorption of Phe_2_ on MMT was a rapid process and a prolonged adsorption time did not significantly affect the adsorption amount. As shown in Figure 3b, more than 85% of the Phe_2_ was desorbed from MMT in 20 min, and the equilibrium state was reached after 30 min. The fast adsorption speed allowed the peptides to rapidly concentrate on MMT from solution, while the rapid desorption was beneficial for the release of peptides.

### 3.2. X-Ray Diffraction Analysis

As shown in Table 1, both of Pro_2_ and Phe_2_ have high adsorption rates on MMT. In order to avoid the influence of phenyl group π-π stacking, we selected Pro_2_ as the tested sample to carry out XRD analysis.

**Figure 4:**
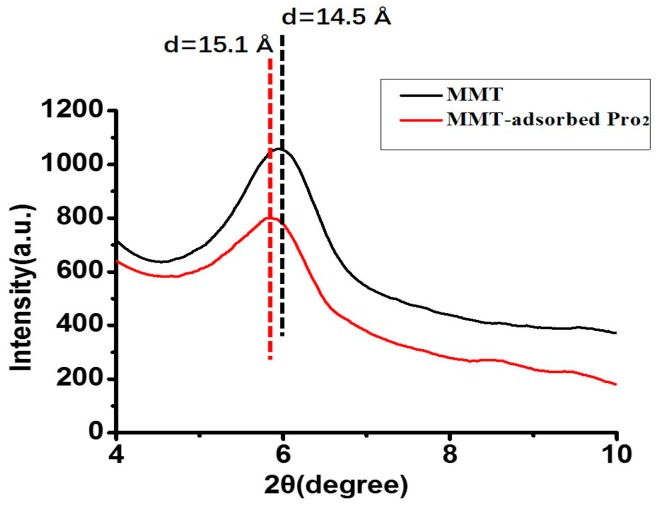
XRD patterns of the initial MMT (black) and Pro_2_ adsorbed on MMT (red).

The XRD pattern of MMT exhibits the (001) characteristic diffraction, which appeared at approximately 6° (2θ) with a d_001_ value of 14.5 Å. In the XRD pattern of MMT with adsorbed Pro_2_, the basal spacing was about 15.1 Å, which was 0.6 Å larger than the initial value (Figure 4). As it was expected that the d_110_ would increase slightly if there was penetration of amino acids or peptides into the interlayer of MMT, these results indicate Pro_2_ was passing into the interlayer of MMT rather than simply undergoing surface adsorption. Because the MMT interlayer was not exposed to UV irradiation directly, it was assumed that the amino acids/peptides sitting into the interlayer were more effectively protected from UV irradiation.

### 3.3. Protective Effect of MMT on Adsorbed Peptides against UV Irradiation

In order to examine whether the adsorption of peptides on MMT can resist photodegradation by prolonged UV irradiation, a total of four sets of experiments were carried out (Table S2). In addition, an extra set of experiments with UV radiation exposure times of 1 and 5 days, respectively, was performed, to explore the influence of illumination time.

**Figure 5:**
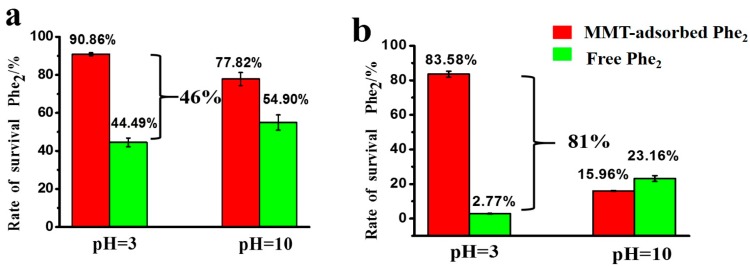
Rate of survival of Phe_2_ after exposure to UV radiation: (**a**) UV exposure time of 1 day. (**b**) UV exposure time of 5 days.

Some preliminary experiments under different conditions (with or without UV irradiation; with or without MMT assistance) were analyzed by HPLC-MS after 1 day or 5 days (Figure S9 in Supporting Information). The results indicate that the UV absorption peak of Phe_2_ decreased gradually with the extension of the exposure time from 1 day to 5 days. After 5 days exposure without MMT, the absorption peak of Phe_2_ in the HPLC spectrum almost disappeared, and there were several new peaks. In contrast, Phe_2_ still remained as a major component if the sample was irradiated with MMT. We are not going to discuss the photochemical reactions of Phe_2_ in this paper, but the characterizations of the new peaks are ongoing works in our lab. As shown in Figure 5a, the survival rates of MMT-adsorbed Phe_2_ were better than that of free Phe_2_ regardless of the pH values. When the samples irradiated for 5 days (Figure 5b), the survival rate of MMT-adsorbed Phe_2_ was 83.6% under acidic condition. The difference in survival rate between MMT-adsorbed Phe_2_ and free Phe_2_ at pH 3 rose from 46% for 1 day exposure to 81% for 5 days exposure. This dramatic increase suggests that the MMT-adsorbed Phe_2_ can effectively resist UV irradiation under acidic conditions with long-term exposure.

**Figure 6:**
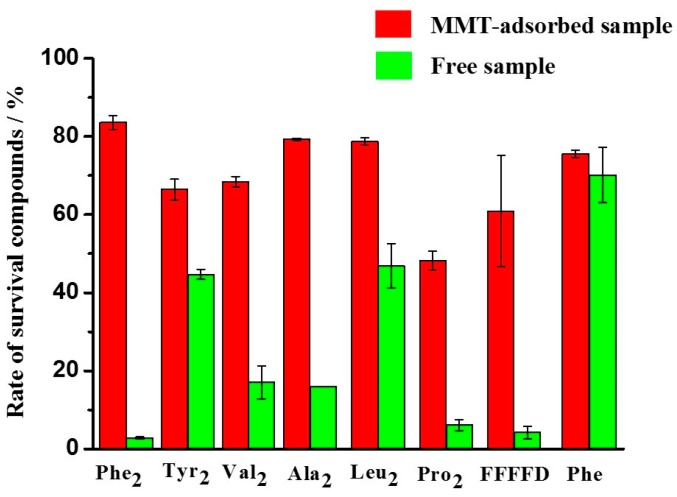
Survival rates of different peptides after exposure to UV radiation for 5 days at pH 3.

Based on these results, we have performed similar experiments on other amino acid/peptides. The results are shown in Figure 6 and Table 2. When comparing the survival rate of MMT-adsorbed peptides and free peptides exposed to UV irradiation for 5 days at pH 3, it was revealed that all the MMT-adsorbed peptides have a higher survival rate than the corresponding free amino acid/peptides (Figure 6). A good protective effect of MMT has been observed on peptides, especially Phe_2_ (Figure 6 and Table 2).

As shown in Table 2, the differences in the survival rate between MMT-adsorbed samples and the corresponding free samples at pH 3 increased with the extension of the exposure time from 1 day to 5 days. In other words, the protective effect of MMT on peptides was more obvious at low pH conditions under longer UV irradiation.

At acidic conditions, Tyr_2_, under UV irradiation for 5 days without MMT, achieved a survival rate of 44.7%. But MMT-absorbed Tyr_2_ had a survival rate of 66.4% after 5 days of UV irradiation. The difference of these two samples was only 21.7%. This indicates that the protective effect of MMT on Tyr_2_ was inconspicuous compared with Phe_2_. Tyr has a similar structure to Phe, except for a phenolic hydroxyl group. We speculated that the strong intermolecular interactions due to the H-bond of the phenolic hydroxyl group may play some roles in resisting UV irradiation, even without the assistance of MMT.

In addition, MMT-adsorbed dipeptides (Ala_2_, Val_2_, Leu_2_ and Pro_2_) were more stable than free dipeptides under UV irradiation for 5 days under acidic conditions (Table 2). The protective effect of MMT on Ala_2_ was the strongest among these four dipeptides, while the weakest protective effect was for Leu_2_. Ala_2_ had the smallest side chain among them, while Leu had the largest one. The differences in amino acid residue side chain may have some effects on MMT’s protective effect on dipeptides.

For all the tested samples, MMT had good adsorption rates of these compounds under acidic conditions, and the MMT-adsorbed samples were protected from UV irradiation which may exist on primitive Earth. This indicates that the MMT-adsorption process would be a way to protect prebiotic organic molecules from photodegradation by UV irradiation

## 4. Discussion

The adsorption properties of different peptides on MMT at different pH values were studied. MMT clay has a high cation-exchange capacity and a large surface area [37], and it shows different adsorption capacities for peptides under different conditions. As shown in Figure 2 and Table 1, the adsorption of peptides on MMT is a pH-dependent process. MMT strongly adsorbs peptides under acidic conditions (e.g., pH = 3), while peptide adsorption on MMT is less efficient under basic conditions (e.g., pH = 10). Different compounds show different adsorption efficiencies. Under the same conditions, Phe has only a 47.5% adsorption rate at pH 3, while the adsorption rate of FFFFD is 99.4%.

The differences in adsorption efficiency are related to the p*K*_a1_ values of the different samples. ChemOffice (Cambridgesoft Corporation, Copyright 1998–2014 PerkinElmer Inc.) was used to predict the p*K*_a1_ values of the tested samples (Table 3). During the adsorption experiments, the pH values of the solutions were adjusted to 3. Most of the chosen compounds have p*K*_a1_ values around 3. Under this condition, around half the carboxyl groups of the peptides on the C-terminal are protonated, making the peptide molecules have a positive charge (the amino group on the N-terminal is already protonated at this pH value). Because the MMT adsorption process is an ion-exchange process, the more positive forms of peptides in solution, the better the MMT adsorption process. This is confirmed by comparing the adsorption rate of Phe (p*K*_a1_ = 2.2, adsorption rate: 47.5%), Phe_2_ (p*K*_a1_ = 2.7, adsorption rate: 97.6%) and FFFFD (p*K*_a1_ = 3.1, adsorption rate: 99.4%). Although the p*K*_a1_ values of Tyr_2_ and Leu_2_ are lower than 3, at this pH value they still have high adsorption rates of 80.3% and 62.1%, respectively (Table 1). These results indicate that there are some other processes that may influence peptide adsorption on MMT, such as hydrogen bonding, dative covalent bonds and dispersive interactions [38].

The process of adsorption/desorption of peptides on MMT is a pH-dependent process. Peptides have a high adsorption rate under acidic conditions. Additionally, the MMT-adsorbed peptides release to the solution when the solution pH value goes to 10. The adsorption of peptides on MMT is also a dynamic process, which can be reversed by adjusting the pH value. At pH 10, most of the peptides adsorbed on MMT can be desorbed (Figure 3b and Table 1).

All the MMT-adsorbed samples had higher survival rates than free samples when they were under UV irradiation for 1 day at pH 3 (Figure 6), and as the UV irradiation time is extended from 1 day to 5 days, the protective effect of MMT on the peptides becomes more obvious. These results show that the adsorption of peptides on MMT can help them resist UV degradation.

In addition, Figure 3 demonstrates that peptide adsorption/desorption on MMT is a rapid equilibrium and can reach the equilibrium point in half an hour. This may be important for chemical evolution. Firstly, peptides can be quickly concentrated on MMT under acidic conditions providing an interface for further peptide evolution. Secondly, after peptides were produced on early Earth, the rapid adsorption of peptides on MMT could protect them from UV decomposition, especially under prolonged irradiation. However, lots of studies have experimentally demonstrated prebiotic chemistry reactions that happened at high pH [39,40,41]. For example, Sakata reported glycine (Gly) dimerized at pH 9.8 in aqueous solution [41]. Ninette and his coworker synthesized peptides starting from *N*-acyl amino acid under mild acidic conditions (pH 5.5–6.5) [42]. Combined with our results, here is a plausible prebiotic scenario for peptide formation. Short peptides are stored by the MMT adsorption process under acidic conditions and protected from photodegradation. Then, the pH value of the environment is increased by geochemical processes (ammonia generation by photoreduction of hydrogen cyanide, etc.); the peptides are able to release into solution and are ready for polymerization into longer peptides.

**Figure 7:**
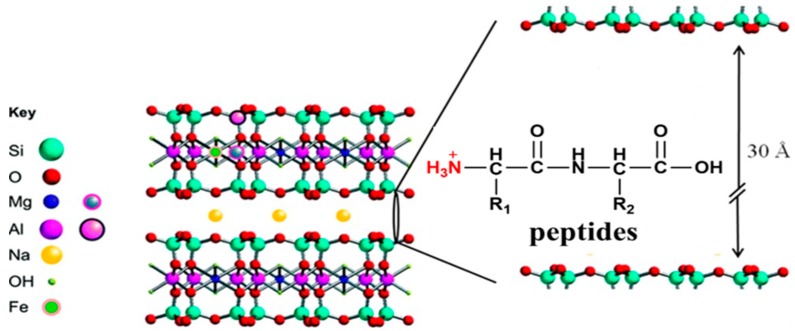
Unit cell of MMT [44] and a possible protection mechanism of MMT for peptides.

Here, we propose a possible protection mechanism of MMT for peptides (Figure 7). First of all, due to the special properties of MMT, the high valence cations (Si^4+^, Al^3+^) in the interlayer of MMT can be replaced by low-valence cations (Mg^2+^, Na^+^), resulting an overall negative charge state. Under acidic conditions (pH 3), peptides are prone to be protonated, resulting in mono/di valence cations. These peptide cations have the same behavior as metal ions, which can replace the high valence cations in the interlayer of MMT. By electrostatic force, the peptide cations can be adsorbed into the interlayer of MMT. Combined with the findings of other reports [43], hydrogen bonding between the protonated amino group (-NH_3_^+^) and the basal oxygen of the interlayer surface would help the adsorption process. Therefore, peptides are not only adsorbed on the surface of the MMT, but also adsorbed into the interlayer, which is not easily exposed to UV irradiation. This means that MMT-adsorbed peptides can become more stable than free peptides under UV irradiation.

## 5. Conclusions

Here, the pH-dependent adsorption of amino acid (Phe) and peptides (Phe_2_, Tyr_2_, Val_2_, Ala_2_, Leu_2_, Pro_2_, FFFFD) on MMT was systematically explored. We found that most of the samples could be efficiently adsorbed on MMT at low pH values. The MMT adsorption rates decrease under higher pH value conditions. Moreover, the adsorption effect is enhanced by the extension of the peptide chain. Furthermore, we found that MMT-adsorbed peptides were protected from UV irradiation. Our results show that MMT-adsorbed peptides are less damaged than free ones when they are exposed to UV irradiation, especially under acidic condition. Through the synergy of many factors, such as electrostatic interaction, ion exchange and hydrogen bonding, amino acids and peptides not only attach on the surface of the MMT, but also adsorb into the interlayer where UV irradiation does not reach easily.

All these findings indicate that peptide is adsorbed on MMT easily under acidic (pH = 3) conditions, which are conducive to protecting biological molecules from UV irradiation under prebiotic conditions. When the environments fluctuate to high pH levels, MMT-adsorbed peptides could be released effectively, ready for further usage (polymerization etc.). This provides a concentration–storage–release cycle of oligopeptides, enabling further polypeptide synthesis from low concentrations of oligopeptides/amino acids. Therefore, pH-dependent adsorption of peptides on MMT may be an important process in the origin of life, which will link to biochemical evolution on early Earth. As a matter of fact, photochemical reactions generally display a dependence on the irradiation wavelength [45,46]. The photo-stability of prebiotic bio-organic molecules at different wavelengths relates to the prebiotic selection of canonical building blocks as well [19]. Hence, it is also worth noting the photo-stability and photochemical behavior of prebiotic bio-organic molecules at multiple wavelengths.

## Figures and Tables

**Table 1:** Adsorption and desorption rate of peptides on MMT under different pH conditions *.

Sample Name	Adsorbed/%		^#^Desorbed/%
	pH 10	pH 3	pH 10
Phe_2_	13.45%	97.57%	88.41%
Tyr_2_	2.09%	80.25%	97.25%
Val_2_	5.14%	62.36%	96.02%
Ala_2_	3.99%	77.04%	96.39%
Leu_2_	2.51%	62.14%	98.23%
Pro_2_	30.53%	90.09%	63.55%
FFFFD	1.81%	99.38%	87.79%
Phe	33.36%	47.46%	65.79%

* Note: Each standard deviation of the data is shown in Table S1. ^#^ The calculation of the desorbed amounts is based on the amount recovered from the adsorbed amount (at pH 3, in middle column).

**Table 2:** Survival rates of peptides under UV irradiation with/without MMT *.

		Items	MMT-Adsorbed Sample				Free Sample			
	Survival Rate/%		pH = 10		pH = 3		pH = 10		pH = 3	
Sample			1 day	5 days	1 day	5 days	1 day	5 days	1 day	5 days
Phe_2_			77.82%	15.96%	90.86%	83.58%	54.90%	23.16%	44.49%	2.77%
Tyr_2_			50.72%	33.29%	79.39%	66.42%	61.55%	41.98%	64.77%	44.69%
Val_2_			84.16%	56.23%	83.74%	68.41%	85.25%	60.47%	76.14%	14.03%
Ala_2_			63.29%	50.76%	87.43%	79.31%	82.76%	19.16%	79.63%	15.92%
Leu_2_			84.55%	27.17%	92.30%	78.61%	87.31%	44.56%	86.72%	42.75%
Pro_2_			18.78%	17.36%	46.57%	45.41%	21.12%	trace	35.72%	6.49%
FFFFD			73.38%	15.45%	80.36%	53.30%	17.87%	3.55%	31.49%	4.27%
Phe			79.87%	64.56%	88.41%	75.52%	7.50%	1.49%	86.83%	70.63%

^*^ Note: Each standard deviation of the data is shown in Table S3.

**Table 3:** The p*K*_a1_ values of different samples.

Entry	Phe	Phe_2_	FFFFD	Tyr_2_	Val_2_	Ala_2_	Leu_2_	Pro_2_
p*K*_a1_	2.207	2.746	3.122	2.562	3.093	3.026	2.961	3.162

## Supplementary Materials

The following are available online at https://www.mdpi.com/2075-1729/10/4/45/s1, Figure S1: (a) LC-MS chromatograms recorded from the standard solution of Phe_2_. (b) Linearity curve of Phe_2_, Figure S2: (a) LC-MS chromatograms recorded from the standard solution of Tyr_2_. (b) Linearity curve of Tyr_2_, Figure S3: (a) LC-MS chromatograms recorded from the standard solution of Val_2_. (b) Linearity curve of Val_2_, Figure S4: (a) LC-MS chromatograms recorded from the standard solution of Ala_2_. (b) Linearity curve of Ala_2_, Figure S5: (a) LC-MS chromatograms recorded from the standard solution of Leu_2_. (b) Linearity curve of Leu_2_, Figure S6: (a) LC-MS chromatograms recorded from the standard solution of Pro_2_. (b) Linearity curve of Pro_2_, Figure S7: (a) LC-MS chromatograms recorded from the standard solution of FFFFD. (b) Linearity curve of FFFFD, Figure S8: (a) LC-MS chromatograms recorded from the standard solution of Phe. (b) Linearity curve of Phe, FigureS9: HPLC chromatograms of Phe_2_ exposed to UV at 254 nm with and without MMT addition for 1 day and 5 days, Table S1: Adsorption and desorption rate of peptides on MMT at different pH for 30 min with standard deviation, Table S2: Experimental grouping designs, Table S3: MMT protection of peptides under UV radiation with standard deviation.

## References

[1] Prins L.J. (2015). Emergence of Complex Chemistry on an Organic Monolayer. Accounts Chem. Res..

[2] Ruiz-Mirazo K., Briones C., De La Escosura A. (2013). Prebiotic Systems Chemistry: New Perspectives for the Origins of Life. Chem. Rev..

[3] Miller S.L., Urey H.C. (1959). Organic compound synthesis on the primitive earth. Science.

[4] Kvenvolden K., Lawless J., Pering K., Peterson E., Flores J., Ponnamperuma C., Kaplan I.R., Moore C. (1970). Evidence for extraterrestrial amino-acids and hydrocarbons in the Murchison meteorite. Nature.

[5] Parker E.T., Zhou M., Burton A.S., Glavin D.P., Dworkin J.P., Krishnamurthy R., Fernández F.M., Bada J.L. (2014). A Plausible Simultaneous Synthesis of Amino Acids and Simple Peptides on the Primordial Earth. Angew. Chem. Int. Ed..

[6] Iqubal M.A., Sharma R., Jheeta S. (2017). Kamaluddin Thermal Condensation of Glycine and Alanine on Metal Ferrite Surface: Primitive Peptide Bond Formation Scenario. Life.

[7] Martin R.B. (1998). Free energies and equilibria of peptide bond hydrolysis and formation. Biopolymers.

[8] Lahav N., White D., Chang S. (1978). Peptide formation in the prebiotic era: Thermal condensation of glycine in fluctuating clay environments. Science.

[9] Rode B.M. (1999). Peptide and the origin of life. Peptides.

[10] Imai E., Honda H., Hatori K., Brack A., Matsuno K. (1999). Elongation of oligopeptides in a simulated submarine hydrothermal system. Science.

[11] Schwendinger M.G., Rode B.M. (1992). Investigations on the mechanism of the salt-induced peptide formation. Orig. life Evol. Biosphere.

[12] Canavelli P., Islam S., Powner M.W. (2019). Peptide ligation by chemoselective aminonitrile coupling in water. Nature.

[13] Cockell C.S., Horneck G. (2001). The history of the UV radiation climate of the earth—Theoretical and space-based observations. Photochem. Photobiol..

[14] Garcia-Pichel F. (1998). Solar ultraviolet and the evolutionary history of cyanobacteria. Orig. Life Evol. Biosph..

[15] Nuevo M., Auger G., Blanot D., d’Hendecourt L. (2008). A detailed study of the amino acids produced from the vacuum UV irradiation of interstellar ice analogs. Orig. Life Evol. Biosph..

[16] Sagan C., Khare B.N. (1971). Long-wavelength ultraviolet photoproduction of amino acids on the primitive Earth. Science.

[17] Powner M.W., Anastasi C., Crowe M.A., Parkes A.L., Raftery J., Sutherland J.D. (2007). On the prebiotic synthesis of ribonucleotides: Photoanomerisation of cytosine nucleosides and nucleotides revisited. ChemBioChem.

[18] Patel B.H., Percivalle C., Ritson D.J., Duffy C.D., Sutherland J.D. (2015). Common origins of RNA, protein and lipid precursors in a cyanosulfidic protometabolism. Nat. Chem..

[19] Janicki M.J., Roberts S.J., Šponer J., Powner M.W., Góra R.W., Szabla R. (2018). Photostability of oxazoline RNA-precursors in UV-rich prebiotic environments. Chem. Commun..

[20] Bonfio C., Valer L., Scintilla S., Shah S., Evans D.J., Jin L., Szostak J.W., Sasselov D.D., Sutherland J.D., Mansy S.S. (2017). UV-light-driven prebiotic synthesis of iron-sulfur clusters. Nat. Chem..

[21] Cockell C.S. (2000). The ultraviolet history of the terrestrial planets—Implications for biological evolution. Planet. Space Sci..

[22] Cleaves H.J., Miller S.L. (1998). Oceanic protection of prebiotic organic compounds from UV radiation. Proc. Natl. Acad. Sci. USA.

[23] Scappini F., Casadei F., Zamboni R., Monti S., Giorgianni P., Capobianco M.L. (2007). Laboratory simulation of UV irradiation from the Sun on amino acids. I: Irradiation of tyrosine. Int. J. Astrobiol..

[24] Scappini F., Capobianco M.L., Casadei F., Zamboni R., Giorgianni P. (2007). Laboratory simulation of UV irradiation from the Sun on amino acids. II. Irradiation of phenylalanine and tryptophan. Int. J. Astrobiol..

[25] Scappini F., Casadei F., Zamboni R., Franchi M., Gallori E., Monti S. (2004). Protective effect of clay minerals on adsorbed nucleic acid against UV radiation: Possible role in the origin of life. Int. J. Astrobiol..

[26] Papke K.G. (1969). Montmorillonite deposits in Nevada. Clays Clay Miner..

[27] Zaia D.A.M. (2004). A review of adsorption of amino acids on minerals: Was it important for origin of life?. Amino Acids.

[28] Kalra S., Pant C.K., Pathak H.D., Mehata M.S. (2003). Studies on the adsorption of peptides of glycine/alanine on montmorillonite clay with or without co-ordinated divalent cations. Colloid Surface A.

[29] Hashizume H. (2015). Adsorption of nucleic acid bases, ribose, and phosphate by some clay minerals. Life.

[30] Ferris J.P., Hagan W.J. (1986). The Adsorption and Reaction of Adenine-Nucleotides on Montmorillonite. Orig. Life Evol. Biosph..

[31] Bernal J.D. (1951). The physical basis of life. Proc. R. Soc. Lond. A.

[32] Boillot F., Chabin A., Bure C., Venet M., Belsky A., Bertrand-Urbaniak M., Delmas A., Brack A., Barbier B. (2002). The Perseus Exobiology mission on MIR: Behaviour of amino acids and peptides in earth orbit. Orig. Life Evol. Biosph..

[33] Scappini F., Capobianco M.L., Casadei F., Zamboni R. (2009). Laboratory simulation of ultraviolet irradiation from the Sun on amino acids. III. irradiation of glycine-tyrosine. Int. J. Astrobiol..

[34] Ma B.G., Chen L., Ji H.F., Chen Z.H., Yang F.R., Wang L., Qu G., Jiang Y.Y., Ji C., Zhang H.Y. (2008). Characters of very ancient proteins. Biochem. Biophys. Res. Commun..

[35] Ranjan S., Sasselov D.D. (2017). Constraints on the Early Terrestrial Surface UV Environment Relevant to Prebiotic Chemistry. Astrobiology.

[36] Ranjan S., Sasselov D.D. (2016). Influence of the UV Environment on the Synthesis of Prebiotic Molecules. Astrobiology.

[37] Dashman T., Stotzky G. (1985). Physical-properties of homoionic montmorillonite and kaolinite complexed with amino-acids and peptides. Soil Biol. Biochem..

[38] Rimola A., Sodupe M., Ugliengo P. (2019). Role of mineral surfaces in prebiotic chemical evolution. in silico quantum mechanical studies. Life.

[39] Holm N.G., Dumont M., Ivarsson M., Konn C. (2006). Alkaline fluid circulation in ultramafic rocks and formationof nucleotide constituents: A hypothesis. Geochem. Trans..

[40] Russell M.J. (2003). The importance of being alkaline. Science.

[41] Sakata K., Kitadai N., Yokoyama T. (2010). Effects of pH and temperature on dimerization rate of glycine: Evaluation of favorable environmental conditions for chemical evolution of life. Geochim. Cosmochim. Acta.

[42] Abou Mrad N., Ajram G., Rossi J.C., Boiteau L., Duvernay F., Pascal R., Danger G. (2017). The Prebiotic C-Terminal Elongation of Peptides Can Be Initiated by N-Carbamoyl Amino Acids. Chem. A Eur. J..

[43] Ramos M.E., Huertas F.J. (2013). Adsorption of glycine on montmorillonite in aqueous solutions. Appl. Clay Sci..

[44] Joshi P.C., Aldersley M.F., Delano J.W., Ferris J.P. (2009). Mechanism of montmorillonite catalysis in the formation of RNA oligomers. J. Am. Chem. Soc..

[45] Todd Z.R., Szabla R., Szostak J.W., Sasselov D.D. (2019). UV photostability of three 2-aminoazoles with key roles in prebiotic chemistry on the early earth. Chem. Commun..

[46] Todd Z.R., Fahrenbach A.C., Magnani C.J., Ranjan S., Bjorkbom A., Szostak J.W., Sasselov D.D. (2018). Solvated-electron production using cyanocuprates is compatible with the UV-environment on a Hadean-Archaean Earth. Chem. Commun..

