# Supplementary material for: pH-Dependent Adsorption of Peptides on Montmorillonite for Resisting UV Irradiation
Source: Life (Basel). 2020 Apr 20;10(4):45. doi: 10.3390/life10040045 (PMC7235719; doi:10.3390/life10040045)
Supplement: Supplementary file 1 [file life-10-00045-s001.pdf]

life

Supporting Information

pH-dependent Adsorption of Peptides on Montmorillonite for Resisting UV irradiation

Rongcan Lin [1,†], Yueqiao Wang [1,†], Xin Li [1], Yan Liu [1,*] and Yufen Zhao [1,2,3]

[1] Department of Chemical Biology, Key Laboratory for Chemical Biology of Fujian Province, College of Chemistry and Chemical Engineering, Xiamen University, Xiamen 361005, China; lyric@stu.xmu.edu.cn (R.L.); qiao510614851@163.com (Y.W.); 13606093461@163.com (X.L.); yfzhao@xmu.edu.cn (Y.Z.)
[2] Institute of Drug Discovery Technology, Ningbo University, Ningbo 315211, China.
[3] Key Laboratory of Bioorganic Phosphorus Chemistry and Chemical Biology (Ministry of Education), Department of Chemistry, Tsinghua University, Beijing 100084, China
[*] Correspondence: stacyliu@xmu.edu.cn
[†] These authors contributed equally to this work
Received: 19 February 2020; Accepted: 17 April 2020; Published: 20 April 2020

Indexes

Figure S1. (a) LC-MS chromatograms recorded from the standard solution of Phe_2. (b) Linearity curve of Phe_2.

Figure S2. (a) LC-MS chromatograms recorded from the standard solution of Tyr_2. (b) Linearity curve of Tyr_2.

Figure S3. (a) LC-MS chromatograms recorded from the standard solution of Val_2. (b) Linearity curve of Val_2.

Figure S4. (a) LC-MS chromatograms recorded from the standard solution of Ala_2. (b) Linearity curve of Ala_2.

Figure S5. (a) LC-MS chromatograms recorded from the standard solution of Leu_2. (b) Linearity curve of Leu_2.

Figure S6. (a) LC-MS chromatograms recorded from the standard solution of Pro_2. (b) Linearity curve of Pro_2.

Figure S7. (a) LC-MS chromatograms recorded from the standard solution of FFFFD. (b) Linearity curve of FFFFD.

Figure S8. (a) LC-MS chromatograms recorded from the standard solution of Phe. (b) Linearity curve of Phe.

Figure S9. HPLC chromatograms of Phe_2 exposed to UV at 254 nm with and

without MMT addition for 1 day and 5 days.

Table S1. Adsorption and desorption rate of peptides on MMT at different pH for 30 min with standard deviation.

Table S2. Experimental grouping designs.

Table S3. MMT protection of peptides under UV radiation with standard deviation.

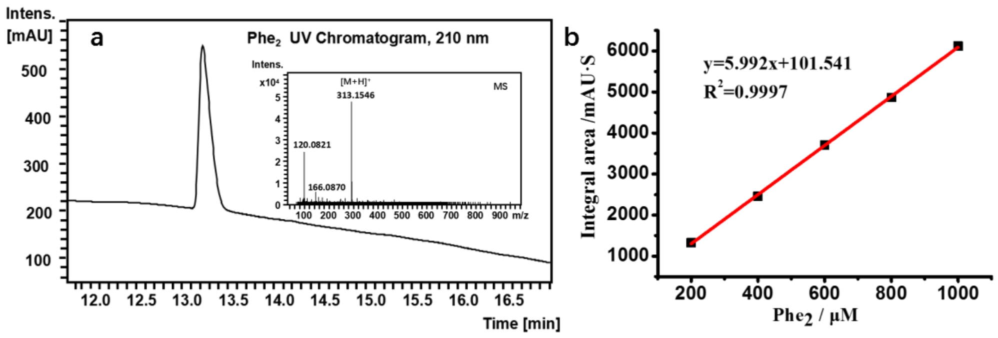

Figure S1. (a) LC-MS chromatograms recorded from the standard solution of Phe_2.

(b) Linearity curve of Phe_2.

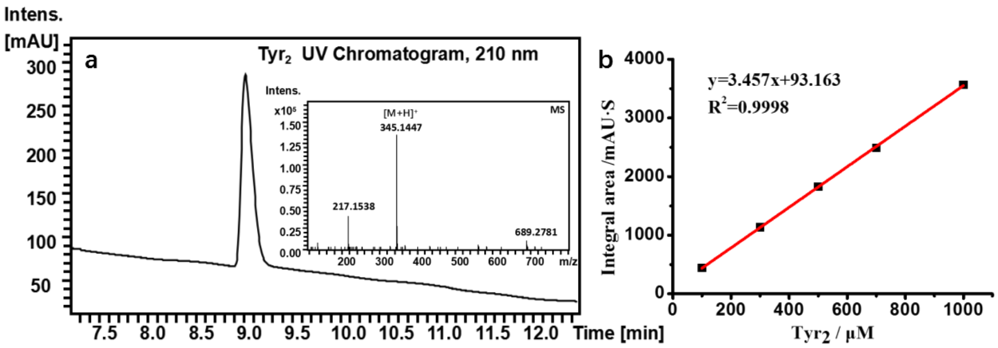

Figure S2. (a) LC-MS chromatograms recorded from the standard solution of Tyr_2.

(b) Linearity curve of Tyr_2.

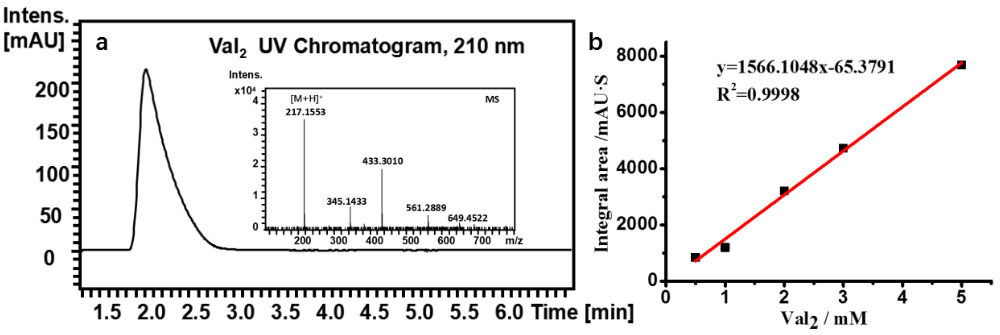

Figure S3. (a) LC-MS chromatograms recorded from the standard solution of Val_2.

(b) Linearity curve of Val_2.

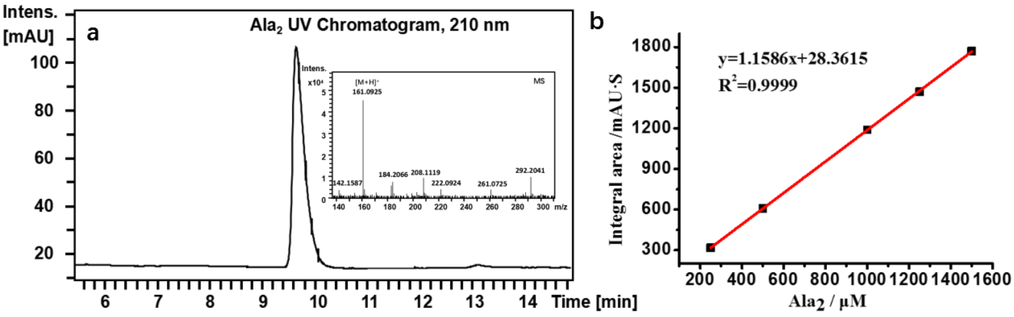

Figure S4. (a) LC-MS chromatograms recorded from the standard solution of Ala_2.

(b) Linearity curve of Ala_2.

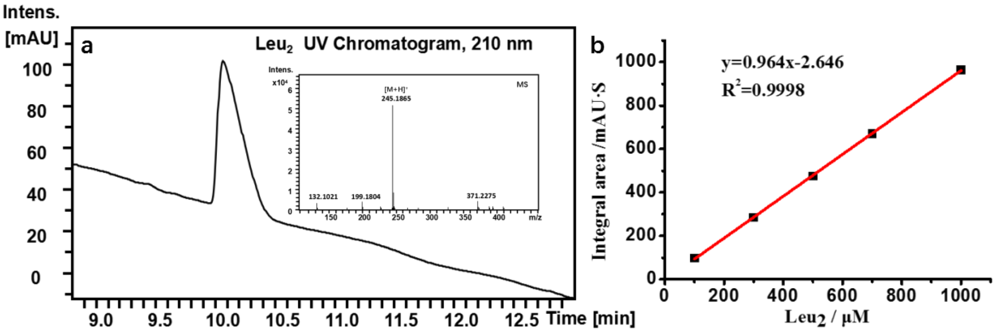

Figure S5. (a) LC-MS chromatograms recorded from the standard solution of Leu_2.

(b) Linearity curve of Leu_2.

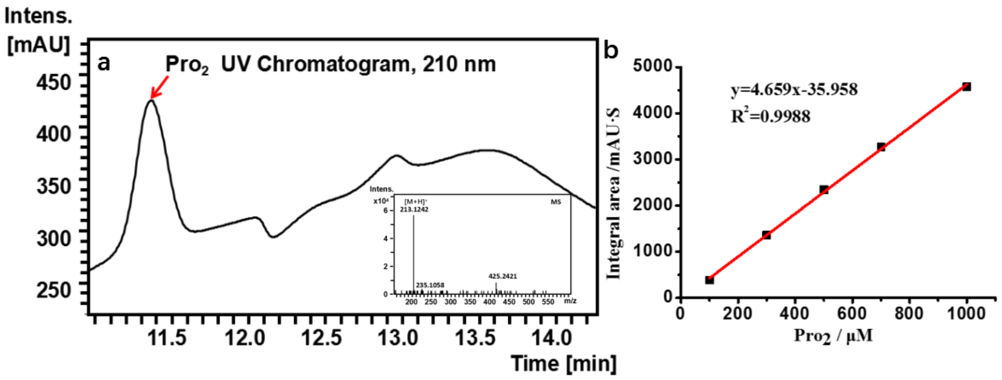

Figure S6. (a) LC-MS chromatograms recorded from the standard solution of Pro_2. (b) Linearity curve of Pro_2.

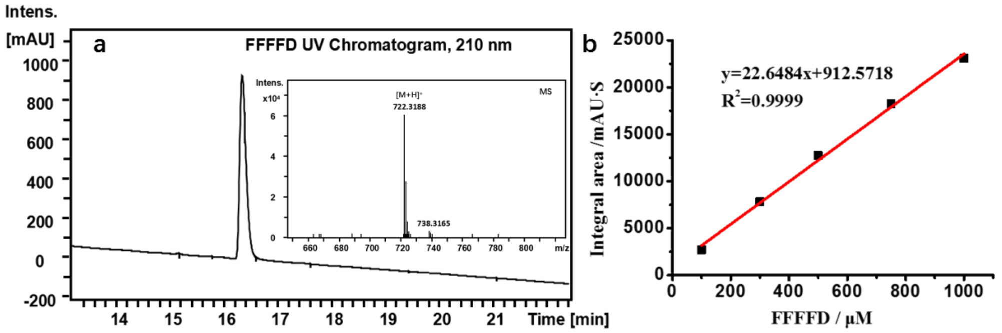

Figure S7. (a) LC-MS chromatograms recorded from the standard solution of FFFFD. (b) Linearity curve of FFFFD.

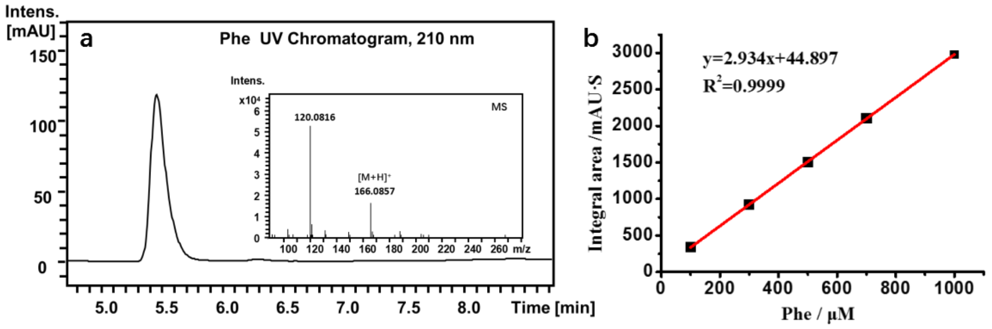

Figure S8. (a) LC-MS chromatograms recorded from the standard solution of Phe. (b) Linearity curve of Phe.

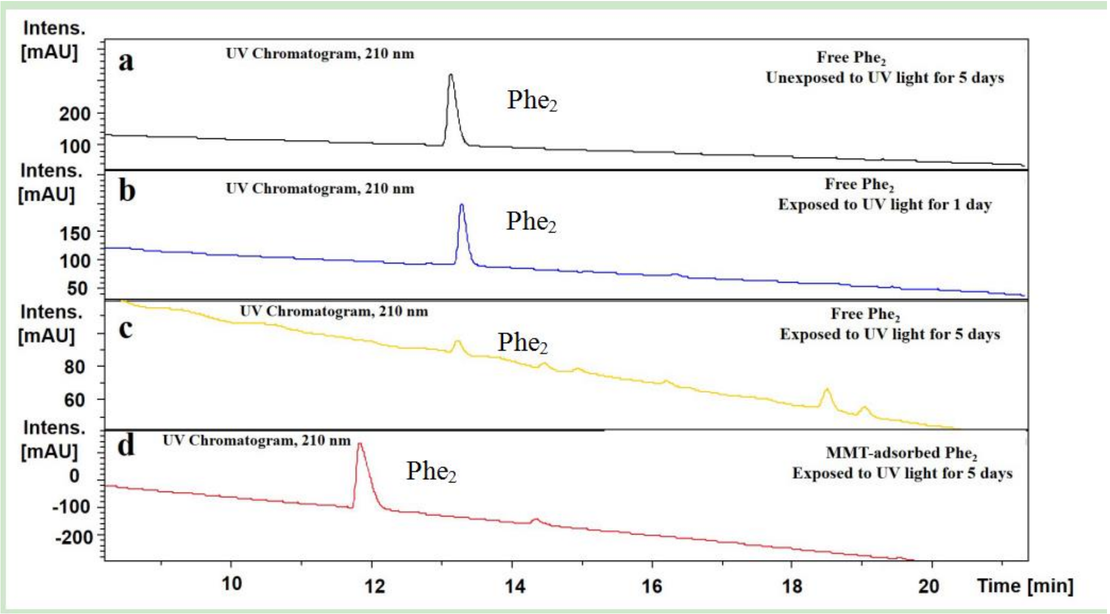

Figure S9. HPLC chromatograms of Phe_2 exposed to UV at 254 nm with and without MMT addition for 1 day and 5 days. (a) The HPLC chromatogram of free Phe_2 unexposed to UV for 5 days. (b) The HPLC chromatogram of free Phe_2 exposed to UV for 1 days. (c) The HPLC chromatogram of free Phe_2 exposed to UV for 5 days. (d) The HPLC chromatogram of MMT-adsorbed Phe_2 exposed to UV for 5 days.

Table S1. Adsorption and desorption rate of peptides on MMT at different pH for 30 min with standard deviation.

Sample name	Adsorption Rate/%		Desorption Rate /%
	pH=10	pH=3	pH=10
**Phe$_2$**	13.45% （0.47）	97.57% （0.17）	88.41% (0.51)
**Tyr$_2$**	2.09% （0.07）	80.25% （0.11）	97.25% (4.08)
**Val$_2$**	5.14% （2.39）	62.36% （1.41）	96.02% （8.41）
**Ala$_2$**	3.99% （4.06）	77.04% （1.18）	96.39% (0.34)
**Leu$_2$**	2.51% (3.56)	62.14% (0.25)	98.23% (0.73)
**Pro$_2$**	30.53% （0.71）	90.09% （0.48）	63.55% （2.98）
**FFFFD**	1.81% （0.66）	99.38% （0.012）	87.79% (0.15)
**Phe**	33.36% （0.63）	47.46% （1.04）	65.79% (1.65)

Note: The numbers in the brackets stand for standard deviation.

Table S2. Experimental grouping designs.

Group	pH=3	pH=10	MMT	Without MMT
1	√		√	
2	√			√
3		√	√	
4		√		√

Table S3. MMT protection of peptides under UV radiation with standard deviation.

Items / Survival Rate/% / Sample	MMT-adsorbed Sample				Free Sample			
	pH=10		pH=3		pH=10		pH=3	
	1 day	5 days	1 day	5 days	1 day	5 days	1 day	5 days
Phe$_2$	77.82% （3.42）	15.96% （0.25）	90.86% （0.78）	83.58% (1.79)	54.9% (4.04)	23.16% (1.72)	44.49% (2.32)	2.77% (0.25)
Tyr$_2$	50.72% （1.69）	33.29% （1.20）	79.39% （0.63）	66.42% (2.67)	61.55% (4.61)	41.98% (3.09)	64.77% (0.33)	44.69% (1.26)
Val$_2$	84.16% （2.08）<br>63.29% （8.26）	56.23% （1.38）<br>50.76% （1.67）	83.74% （0.66）	68.41% (1.34)	85.25% (2.37)	60.47% (0.87)	76.14% (2.62)	14.03% (4.22)
Ala$_2$	84.55% (1.85)	27.17% （1.85）	87.43% （3.52）	79.31% (0.29)	82.76% (3.73)	19.16% (3.46)	79.63% (1.70)	15.92% (0.02)
Leu$_2$	18.78% （1.59）	17.36% （1.58）	92.3% （3.89）	78.61% (0.99)	87.31% (4.89)	44.56% (3.31)	86.72% (5.54)	42.75% (5.61)
Pro$_2$	73.38% （0.76）	15.45% （3.45）	46.57% （0.65）	45.41% (2.45)	21.12% (1.16)	trace	35.72% (9.39)	6.49% (1.36)
FFFFD	79.87% （1.33）	64.56% （3.43）	80.36% （6.26）	53.3% (14.20)	17.87% (8.31)	3.55% (1.48)	31.49% (3.53)	4.27% (1.60)
Phe			88.41% （0.58）	75.52% (0.93)	7.5% (0.33)	1.49% (0.56)	86.83% (3.67)	70.63% (7.12)

Note: The numbers in the brackets stand for standard deviation.
